# National patterns of physician management of sleep apnea and treatment among patients with hypertension

**DOI:** 10.1371/journal.pone.0196981

**Published:** 2018-05-23

**Authors:** Rebecca Robbins, Azizi Seixas, Girardin Jean-Louis, Sairam Parthasarathy, David M. Rapoport, Gbenga Ogedegbe, Joseph A. Ladapo

**Affiliations:** 1 Department of Population Health, NYU School of Medicine, New York, New York, United States of America; 2 Department of Medicine, University of Arizona, Tuscon, Arizona, United States of America; 3 Division of Pulmonary, Critical Care and Sleep Medicine, Icahn School of Medicine at Mount Sinai, New York, New York, United States of America; 4 Division of General Internal Medicine and Health Services Research, David Geffen School of Medicine at UCLA, Los Angeles, California, United States of America; University of Rome Tor Vergata, ITALY

## Abstract

**Study objectives:**

Sleep apnea is associated with hypertension, and treatment may improve outcomes. We examine national burden of sleep apnea, rates of sleep apnea treatment, and whether racial/ethnic disparities exist among patients with hypertension.

**Methods:**

Data from the National Ambulatory Medical Care Survey/National Hospital Ambulatory Medical Care Survey (NAMCS/NHAMCS), 2005–2012, were analyzed (N = 417,950). We identified hypertension patient visits where sleep apnea diagnosis or complaint was recorded. Primary outcome measures were sleep study, medication, or behavioral therapy (diet, weight loss, or exercise counseling). We used multivariate logistic regression to examine treatment by demographic/clinical factors.

**Results:**

Among patients with hypertension, sleep apnea was identified in 11.2-per-1,000 visits. Overall, patients with hypertension and a sleep disorder were referred for sleep study in 14.4% of visits, prescribed sleep medication in 11.2% of visits, and offered behavioral therapy in 34.8% of visits. Adjusted analyses show behavioral therapy more likely to be provided to obese patients than normal/overweight (OR = 4.96, 95%CI[2.93–8.38]), but less likely to be provided to smokers than nonsmokers (OR = 0.54, 95%CI[0.32–0.93]). Non-Hispanic blacks were less likely to receive medications than non-Hispanic whites (OR = 0.19, 95% CI[0.06–0.65]).

**Conclusions:**

In the U.S., sleep apnea were observed in a small proportion of hypertension visits, a population at high-risk for the disorder. One explanation for the low prevalence of sleep apnea observed in this patient population at high risk for the disorder is under-diagnosis of sleep related breathing disorders. Behavioral therapy was underutilized, and non-Hispanic Blacks were less likely to receive medications than non-Hispanic Whites.

## Introduction

Sleep apnea is common among patients with cardiovascular disease (CVD),[1] particularly hypertension.[2–8] [9] This can be explained in part by the observation that hypertension and sleep apnea share common pathophysiological risk factors that exacerbate both conditions.[10] Sleep apnea is a treatable sleep disorder.[11] Unfortunately, while others have called for increased attention to diagnosing and treating sleep apnea among hypertension patients,[12] we know little about physician management of hypertensive patients presenting with sleep apnea in national samples.

There is growing epidemiological evidence supporting a relationship between sleep apnea and hypertension. Sleep apnea is associated with increased mortality and both hypertension and pulmonary hypertension, in addition to coronary artery disease (CAD), cardiac arrhythmias, congestive heart failure (CHF), and stroke.[11] Previous analysis utilizing the National Ambulatory Medical Care Survey has examined characteristics of sleep-related medical visits in the general population (e.g., prevalence of sleep difficulty, and physician management),[13] yet we know little about trends pertaining to sleep apnea prevalence or treatment among higher-risk patients with hypertension.

In addition to the link between hypertension and sleep, evidence suggests sleep apnea is undertreated in the general population.[14] According to some estimates, sleep apnea affects between 9 and 24 percent of adults in the US,[10,15] but only 2 percent report a diagnosis.[16] Particularly concerning are even lower rates of diagnosis among minority populations who suffer a disproportionately higher burden of hypertension[17] and sleep apnea.[18,19] Blacks in particular are at greater risk of hypertension,[17] disrupted sleep,[19] and sleep apnea[18] than other racial or ethnic groups. We know little about how sleep apnea diagnosis or physician management approaches differ across racial or ethnic groups with cardiovascular risk factors.

Clinical guidelines for the treatment of sleep apnea most often includes a sleep study, and treatment using continuous positive airway pressure (CPAP).[20] Meta-analysis shows an association between use of CPAP in the treatment of OSA and a modest reduction in blood pressure.[21] Other, behavioral and lifestyle treatments that have been explored for the treatment of OSA. Meta-analysis has found behavioral therapy, such as weight loss, to be effective for reduction in OSA parameters.[22] We examine sleep apnea, as reported by patients or diagnosed by physicians, and physician management of sleep apnea, specifically, provision of a sleep study and behavioral treatment and therapy (e.g., weight loss, exercise) among individuals with hypertension. We also examine physician provision of medication to patients with sleep apnea, because some physicians may use sedative agents as a short-term strategy to improve compliance with CPAP[23–25] or prescribe them to patients with comorbid psychiatric illness,[26] though these medications are generally avoided in this population.[27]

National patterns of diagnosis and treatment of sleep apnea in a high-risk population (hypertension patients) has not been studied. In addition, we do not know whether health disparities exist in physician management of such conditions. To better understand sleep apnea prevalence and management in a high-risk population of patients with hypertension, we utilize nationally representative data to examine sleep apnea diagnosis and management during their ambulatory visits. Further, we also evaluated whether racial/ethnic differences exist in physician management among patients with hypertension.

## Methods

### Data and study population

We analyzed data from the 2005–2012 National Ambulatory Medical Care Survey (NAMCS) and National Hospital Ambulatory Medical Care Survey (NHAMCS), nationally representative surveys of adults seeking ambulatory care. Our methods for analyzing NAMCS/NHAMCS data were similar to those we have described in detail previously.[28] Specifically, we included all visits to office-based physicians and hospital-based outpatient clinics by adults 18 years of age or older (N = 417,950 adult visits). NAMCS and NHAMCS are conducted annually by the National Center for Health Statistics (NCHS) and the Centers for Disease Control and Prevention using a nationally representative sample of visits to office-based physicians, hospital-based outpatient clinics, and emergency departments in the United States. For the NAMCS, each physician is randomly assigned to a 1-week reporting period during which a random sample of visits are surveyed systematically. NCHS staff collect data on patients’ symptoms, comorbidity, and demographic characteristics; physicians’ diagnoses; medications ordered or provided; and medical services provided. For the NHAMCS, a systematic random sample of patient visits in selected non-institutional general and short-stay hospitals are surveyed randomly during a assigned 4-week reporting period. The data on patient and provider characteristics collected for NHAMCS are comparable to those collected in the NAMCS.

Data on outpatient hospital departments and community health centers from the NHAMCS were unavailable in 2012 at the time of our analysis, but the majority of ambulatory care is performed in office-based visits and captured by the NAMCS (93% of visits during 2006–2011 occurred in the office rather than hospital outpatient departments, and 99% of office visits occurred outside of community health centers). In sensitivity analyses, we evaluated the effect of removing hospital outpatient departments and community health centers from our estimates of visit and treatment rates, and found that their omission changed our estimated rates by a relative amount of less than 1–2% for most measures. However, we adjusted for the absence of these two care sites in our regression analyses.

The NAMCS and NHAMCS intake materials allow physicians and staff to record up to three reasons for each visit and three diagnoses related to the visit, in addition to capturing several other major comorbid diagnoses (coded by NCHS staff using the International Classification of Diseases, Ninth Revision, Clinical Modification [ICD-9-CM]). From 2005 to 2012, the physician and hospital/outpatient clinic response rates in the NAMCS and NHAMCS ranged from 54% to 73% (except in 2012, when NAMCS response was 39%) and 80% to 95%, respectively, and item nonresponse rates were generally 5% or less in both surveys.

### Study population and measures

We identified patients with hypertension who reported sleep apnea as a reason for visit or for whom sleep apnea was a visit diagnosis. Diagnosis codes and reason for visit codes were derived from a prior study.[13] We examined physician decision-making and treatment patterns for patients with hypertension and sleep apnea using (1) ICD-9 procedure codes for sleep studies[29] (polysomnography and multiple sleep latency test) as previously reported,[13] (2) Multum Lexicon drug codes and therapeutic drug categories and NCHS generic codes for benzodiazepines and non-benzodiazepine sleep aids,[13] (3) positive airway pressure (PAP) treatment, and (4) behavioral therapy (diet, weight loss, or exercise counseling), as reported by physicians during the office visit.[13,30]

### Other measures

To examine factors associated with diagnostic and treatment patterns, we extracted information on demographic characteristics including age, sex, race/ethnicity, insurance (private, Medicare, Medicaid, self-pay/no-charge, and other/unknown), US census region (Northeast, Midwest, South, and West), and urban or rural setting. We characterized patients as non-Hispanic white, non-Hispanic black, Hispanic, or other/unknown race. We also adjusted for the presence of other risk factors associated with diagnostic testing, including obesity, smoking, and diabetes,[31] along with depression, an emerging risk factor.[32,33]

### Statistical analysis

We used summary statistics to estimate the prevalence of sleep apnea and management patterns among patients with hypertension. We also performed multivariate logistic regressions to assess treatment differences while adjusting for patients’ clinical risk factors and demographic characteristics, insurance status, geographical region, and setting (urban or rural). This regression model excluded 4% of the patient population because these patients reported both sleep apnea and insomnia during the same ambulatory care visit, whereas the diagnoses were independently included as model covariates. All analyses accounted for the complex sampling design of the NAMCS and NHAMCS and were performed using Stata, version 14 (StataCorp, Inc. College Station, TX).

## Results

### Patient characteristics and prevalence of sleep apnea

From 2005–2012, patients with hypertension in the United States had an annual average of 3.3 million ambulatory visits for sleep apnea. Most visits were made by older patients (51.4% between ages 45–64 years) and non-Hispanic white (51.4%). Most had private health insurance (52.9%), followed by Medicare (33.2%). Of the hypertension risk factors we assessed, including the emerging risk factor for depression,[32,33] obesity was the most common (37.8%). Among other CVD comorbidities in the population, CAD was most prevalent (9.6%) (see Table 1).

**Table 1:** Characteristics of patients with hypertension and sleep apnea (either as a reason for visit or visit diagnosis) seeing physicians for ambulatory care in the U.S., 2005–2012.

*Characteristic*	*Unweighted No*.	*Weighted Annual No*.	Percent %*	*Standard Error*
Visits	2,612	3,047,801	100.0	0.0
Age, yrs.				
18–44	472	437,304	14.3	1.6
45–64	1,405	1,648,124	54.1	2.0
≧65	735	962,373	31.6	2.0
Sex				
Female	1,142	1,262,190	41.4	2.1
Male	1,470	1,785,611	58.6	2.1
Race/Ethnicity				
Non-Hispanic white	1,392	1,568,021	51.4	3.4
Non-Hispanic black	296	280,104	9.2	2.9
Hispanic	128	203,026	6.7	1.4
Other/unknown	796	996,649	32.7	4.5
Insurance				
Private	1,315	1,610,877	52.9	2.8
Medicare	785	1,010,723	33.2	2.2
Medicaid	278	217,855	7.1	1.2
Other/unknown	159	169,868	5.6	1.1
Uninsured	159	169,868	5.6	1.1
Region				
Northeast	316	356,737	11.7	2.0
Midwest	737	579,886	19.0	2.8
South	1,109	1,370,669	45.0	5.8
West	450	740,507	24.3	4.4
Cardiovascular Risk factors				
Obesity	1,068	1,151,225	37.8	3.7
Smoker	326	366,898	12.0	1.3
Diabetes	779	934,297	30.7	2.2
Depression	518	549,324	18.0	1.9
Comorbid Cardiovascular Disease				
Coronary Artery Disease	210	291,636	9.6	1.4
Congestive Heart Failure	140	177,701	5.8	1.4
Stroke	84	112,959	3.7	0.9

* Percents represent the proportion of patients with sleep apnea within demographic category

### Treatment patterns for sleep apnea

The prevalence of referrals for sleep studies, prescriptions for sleep medications, and referrals for behavioral therapy during ambulatory visits among patients with hypertension are shown in Table 2. Overall, patients with hypertension and sleep apnea were referred for a sleep study in 14.4% of visits, prescribed sleep medications in 12.9% of visits, and offered behavioral therapy in 34.8% of visits.

**Table 2:** Physician management of patients with hypertension and sleep apnea in the U.S., 2005–2012.

Characteristic	*Weighted Annual No*.	*Percent*, *%*	*Standard Error*
Any sleep study	440,376	14.4	1.9
Polysomnography sleep study	392,103	12.9	1.8
Multiple sleep latency test	49,410	1.6	0.5
Prescriptions for Sleep Medications	340,538	11.2	1.5
Benzodiazepine	234,755	7.7	1.4
Nonbenzodiazepine	191,048	6.3	1.1
Behavioral Therapy (Diet, Weight Reduction, Exercise)	1,059,685	34.8	2.7

### Differences in treatment patterns by demographic and clinical characteristics

In multivariate regression analyses, we found that behavioral therapy was more likely to be offered to patients who were obese compared with normal and overweight patients (OR = 4.96, 95% CI: 2.93–8.38), but less likely to be provided to smokers compared with nonsmokers (OR = 0.54, 95% CI: 0.32–0.93). We found racial/ethnic differences in prescription of sleep medications, such that non-Hispanic blacks were less likely than non-Hispanic whites to be prescribed sleep medications (OR = 0.19, 95% CI: 0.06–0.65) as were Other/unknown OR = 0.53, 95% CI: 0.28–1.00). In addition, patients with stroke were less likely than those with hypertension to be prescribed sleep medication (OR = 0.00, 95% CI: 0.00–0.01). There were no racial/ethnic differences in rates of referrals for sleep studies (see Table 3).

**Table 3:** Regression examining disparities in physician treatment of sleep apnea in patients with hypertension seeing physicians for ambulatory care in the U.S., 2005–2012.

Variable	Any Sleep Study		Any Medication		Behavioral Treatment	
	*OR (95% CI)*	*P-Value*	*OR (95% CI)*	*P-Value*	*OR (95% CI)*	*P-Value*
Race/Ethnicity (Reference: White)						
Non-Hispanic black	1.33 (0.59–3.03)	0.493	0.19 (0.06–0.65)	<0.01	0.77 (0.46–1.31)	0.337
Hispanic	0.73 (0.26–2.11)	0.564	0.35 (0.08–1.47)	0.151	1.85 (0.78–4.40)	0.161
Other/unknown	0.87 (0.51–1.49)	0.611	0.53 (0.28–1.00)	0.049	0.70 (0.41–1.19)	0.183
Age (Reference: 18-44yrs)						
45–64	0.80 (0.36–1.75)	0.571	1.81 (0.74–4.46)	0.196	1.01 (0.51–1.98)	0.980
≧65	0.76 (0.24–2.37)	0.637	0.55 (0.14–2.12)	0.382	0.65 (0.29–1.45)	0.294
Sex (Reference: Males)						
Female	1.30 (0.76–2.22)	0.337	1.47 (0.70–3.11)	0.307	1.27 (0.84–1.92)	0.252
Insurance (Reference: Private)						
Medicare	0.50 (0.20–1.21)	0.123	1.77 (0.80–3.91)	0.157	0.90 (0.53–1.54)	0.705
Medicaid	0.46 (0.15–1.40)	0.172	1.57 (0.58–4.25)	0.377	0.77 (0.36–1.68)	0.515
Other/unknown	0.92 (0.37–2.26)	0.851	0.49 (0.16–1.52)	0.217	0.5 (0.19–1.32)	0.161
Uninsured	0.39 (0.05–2.70)	0.337	1.86 (0.38–9.14)	0.446	0.91 (0.32–2.54)	0.853
Location (Reference: Urban)						
Rural	1.10 (0.39–3.08)	0.853	0.68 (0.26–1.72)	0.412	1.36 (0.78–2.38)	0.272
Cardiovascular Risk Factors†						
Obesity	1.15 (0.71–1.87)	0.578	0.77 (0.49–1.21)	0.252	4.96 (2.93–8.38)	<0.001
Smoker	0.55 (0.26–1.15)	0.110	1.03 (0.36–2.95)	0.952	0.54 (0.32–0.93)	0.026
Diabetes	0.94 (0.55–1.61)	0.833	0.89 (0.50–1.60)	0.709	0.87 (0.56–1.36)	0.538
Depression	1.31 (0.79–2.17)	0.302	1.51 (0.76–3.03)	0.242	1.13 (0.72–1.78)	0.586
Comorbid Cardiovascular Diseases						
Coronary Artery Disease	1.69 (0.58–4.91)	0.332	0.28 (0.07–1.06)	0.061	0.84 (0.39–1.80)	0.657
Congestive Heart Failure	1.39 (0.25–7.62)	0.702	1.42 (0.37–5.38)	0.607	1.77 (0.70–4.45)	0.228
Stroke	1.86 (0.36–9.65)	0.460	0.00 (0.00–0.01)	<0.001	0.94 (0.35–2.54)	0.906
Two or More Cardiovascular Diseases*	0.77 (0.22–2.73)	0.686	0.22 (0.03–1.69)	0.145	0.66 (0.22–1.98)	0.460

^†^ Reference group comprises patients without the stated cardiovascular risk factor.

* Patients with two or more cardiovascular diseases (Coronary Artery Disease, Congestive Heart Failure, or Stroke).

## Discussion

In a nationally representative survey of ambulatory medical care visits in the United States, we found a low prevalence of sleep apnea among patients with hypertension (11.1 per 1,000 visits). Treatment for sleep apnea among patients with hypertension included a sleep study in 14.4% of visits, sleep medication in 11.2% of visits, and behavioral therapy in 34.8% of visits. We also found that non-Hispanic blacks and Other/unknown were less likely than non-Hispanic Whites to receive a sleep medication prescription during their visit.

Prevalence estimates of sleep apnea among the general population in the U.S. are as high as 24%.[10] Prevalence of sleep apnea in ambulatory visits was far below these population-level estimates, despite assessing a sub-population at risk for sleep apnea. This finding is consistent with other research showing that sleep apnea is underdiagnosed, which bears particular clinical importance for patients with hypertension.[2,6] Unfortunately, our findings and others support concerns about under-diagnosis of sleep apnea among a population that is at high risk for the disorder.

Although counseling patients about healthy lifestyle behaviors is recommended by professional societies for those with chronic diseases including hypertension and sleep apnea,[34] we found in approximately one third of cases, physicians provide behavioral therapy (weight loss and physical activity counseling) to patients. Our finding underscores a need for assessing efficacy of behavioral methods in the treatment of patients with hypertension and sleep apnea.[35] It remains unclear why behavioral treatment is administered in only one third of cases, or what constitutes effective components of behavioral approaches to treatment. Future research may explore possible reasons for relatively low administration of behavioral treatment. The importance of effective therapy for patients with sleep apnea is further underscored by evidence indicating that sleep apnea may increase the risk of cognitive impairment.[36] For example, sleep apnea is more prevalent among patients with Alzheimer’s disease. In addition, one study showed that improvement of sleep apnea symptoms was associated with higher performance in verbal memory tasks.[37]

Also noteworthy was the modest prevalence of sleep medication. Sleep medications have been linked to adverse health outcomes (toxicity, mortality risk),[38,39] so while it is positive that low prevalence was observed in the current study, other analyses with NAMCS data found between 20% and 80% of patients with sleep apnea receive medication. Results of the current study found relatively low prevalence of medication.

We found little evidence of racial/ethnic differences in physicians’ management of patients with sleep apnea. However, non-Hispanic blacks and other/unknown racial/ethnic groups had lower odds of receiving sleep medications than non-Hispanic whites, but no other comparisons across treatment by race/ethnicity were significant. While there is a well-established literature documenting differences hypertension, sleep apnea, and risk for sleep apnea, physician behavior may not be a substantial contributor to these differences.[40,41] The noted racial/ethnic difference in sleep medication use could be in part due to differences in coverage offered by patient health insurance plans (which we did not control for because these data were unavailable) or differences in the efficacy of screening procedures that could be rooted in cultural or linguistic differences.[42] Our study also shows a sleep study was most common among sleep apnea, as would be expected given sleep medicine guidelines recommend polysomnography for evaluation of sleep apnea.[43]

### Implications

The current study found relatively low prevalence of sleep apnea in a population at high risk for the disorder: hypertensive patients. One implication of these findings is that sleep apnea merits greater attention in patient-physician interactions. It is possible that patients are underreporting sleep apnea symptoms to their physicians, or that physicians are failing to inquire about sleep apnea symptoms or recognize the presence of sleep apnea symptomology among at-risk patients. In a small, single center study, investigators previously reported that only 4% of sleep-center patients were referred after a clinician elicited a history of sleep-related symptoms.[44] Rather, the majority of patients were referred to a sleep center because they complained about sleep-related problems. Methods for improving screening approaches are needed, and existing suggestions include a simple 2-question screening test as part of the review of systems[45] or an informatics-based approach.[46] This is particularly important given the attributable risk associated with sleep apnea among patients with cardiovascular disease. In addition, behavioral therapy was found to be reported in approximately one third of patients reporting sleep apnea. Future research might examine reasons this prevalence is not higher, and what components of behavioral therapy are most effective.

### Limitations

We should note the following limitations of our findings. NAMCS data provide a limited amount of clinical information for each patient visit. For example, a physician is only able to identify a limited number of comorbidity and symptoms for each visit. For patients with multiple diseases and symptoms, this represents a barrier to a full view of diagnostic and treatment decisions, particularly in chronic disease management. In addition, the data did not include details on whether the decision to refer a patient for a sleep study was based on symptoms, anthropometric parameters, resistant hypertension, or non-dipping blood pressure patterns. We also did not have data on diastolic hypertension or treatment-resistant hypertension, which are more common among patients with sleep apnea. Another important limitation is that the survey does not provide longitudinal follow-up for each patient. Thus, inferences about patterns of care are based on cross-sectional data of diagnostic and treatment decisions instead, and outcome data are unavailable. Also, our study focused narrowly on sleep apnea without considering other sleep disorders. There is a literature to demonstrate a relationship between hypertension, sleep apnea, and other sleep disorders such as periodic limb movements.[36] Finally, our findings could be subject to errors or anomalies in NAMCS/NHAMCS data collection and reporting.

### Conclusion

In conclusion, the rate of sleep apnea observed among patients with hypertension is low, and behavioral therapy is provided to a minority of these patients during sleep apnea-related ambulatory care visits. Racial/ethnic differences in physician management are uncommon. Future research should examine whether efforts to increase recognition of sleep apnea among patients with hypertension affect patterns of behavioral and pharmacological treatment among these patients—along with potential effects on racial/ethnic differences, such as those related to the use of sleep medication.
